# *Pneumocystis jirovecii* pneumonia after CD4+ T‐cell recovery subsequent to CD19‐targeted chimeric antigen receptor T‐cell therapy: A case report and brief review of literature

**DOI:** 10.1002/cnr2.1885

**Published:** 2023-08-10

**Authors:** Kento Kawata, Haruko Shima, Masayoshi Shinjoh, Fumito Yamazaki, Takumi Kurosawa, Mizuki Yaginuma, Hiroshi Takada, Hiroyuki Shimada

**Affiliations:** ^1^ Department of Pediatrics Keio University School of Medicine Tokyo Japan

**Keywords:** B‐cell aplasia, CAR‐T cell therapy, CD4+ T‐cell, hemophagocytic lymphohistiocytosis, pneumocystis pneumonia, relapsed B‐ALL

## Abstract

**Background:**

CD19‐targeted chimeric antigen receptor (CAR)‐T cell therapy involves administration of patient‐derived T cells that target B cells, resulting in B‐cell depletion and aplasia. In immunity against *Pneumocystis jirovecii* (*Pj*), CD4+ T cells and, more recently, B cells, are generally considered important. Antigen presentation by B cells to CD4+ T cells is particularly important. Trimethoprim‐sulfamethoxazole (TMP/SMX) for *Pj* pneumonia (PJP) prophylaxis is generally discontinued when the CD4+ T‐cell count is >200/μL. Here we report the first case, to our knowledge, of PJP in a patient with a CD4+ T cell count of >200/μL after CAR‐T cell therapy.

**Case:**

A 14‐year‐old girl developed hemophagocytic lymphohistiocytosis (HLH) after cord blood transplantation (CBT) for relapsed precursor B‐cell acute lymphoblastic leukemia (B‐ALL). Twenty‐one months after CBT, she was diagnosed with combined second relapse in the bone marrow and central nervous system. The patient was treated with CD19‐targeted CAR‐T cell therapy for the relapse. After CAR‐T cell therapy, the patient remained in remission and continued to receive TMP/SMX for PJP prophylaxis. Seven months after CAR‐T cell therapy, CD4+ T cells recovered and TMP/SMX was discontinued. The B‐cell aplasia persisted. Ten months after CAR‐T cell therapy, the patient developed PJP. The patient was also considered to have macrophage hyperactivation at the onset of PJP. Treatment with immunoglobulin, TMP/SMX, and prednisolone was initiated, and the patient's symptoms rapidly ameliorated.

**Conclusion:**

The patient in the present case developed PJP despite a CD4+ T‐cell count of >200/μL after CAR‐T cell therapy, probably because of inadequate CD4+ T‐cell activation caused by B‐cell depletion after CAR‐T cell therapy and repeated abnormal macrophage immune responses after CBT. It is important to determine the duration of TMP/SMX for prophylaxis after CAR‐T cell therapy according to each case, as well as the CD4+ T‐cell count.

## 1 INTRODUCTION

CD19‐targeted chimeric antigen receptor (CAR)‐T cell therapy involves administration of patient‐derived T cells that target B cells, resulting in B‐cell depletion and aplasia. Various infections can occur after CAR‐T cell therapy. Among these, fungal infections are commonly caused by invasive aspergillosis and candidiasis; however, *Pneumocystis jirovecii* pneumonia (PJP) is a rare complication.^1^, ^2^ Although the immunological mechanism in PJP remains unclear, CD4+ T cells are generally given importance because of their prevalence in patients with acquired immunodeficiency syndrome (AIDS), which is characterized by CD4+ T‐cell depletion.^3^ Of late, B cells have also been considered important in the immune response to *Pneumocystis jirovecii* (*Pj*), and there have been reports of PJP in patients treated with only rituximab, which causes B‐cell dysfunction, and patients with X‐linked agammaglobulinemia, which causes B‐cell aplasia.^4^, ^5^, ^6^, ^7^, ^8^, ^9^ After CAR‐T cell therapy, trimethoprim‐sulfamethoxazole (TMP/SMX) for PJP prophylaxis is usually discontinued within 6 months to 1 year or when the CD4+ T‐cell count is >200/μL.^10^, ^11^ Here we report the first case, to our knowledge, of PJP in a patient with a CD4+ T‐cell count of >200/μL after CD19‐targeted CAR‐T cell therapy. This case is unique because B‐cell aplasia after CAR‐T cell therapy may have contributed to the development of PJP.

## 2 CASE

**FIGURE 1:**
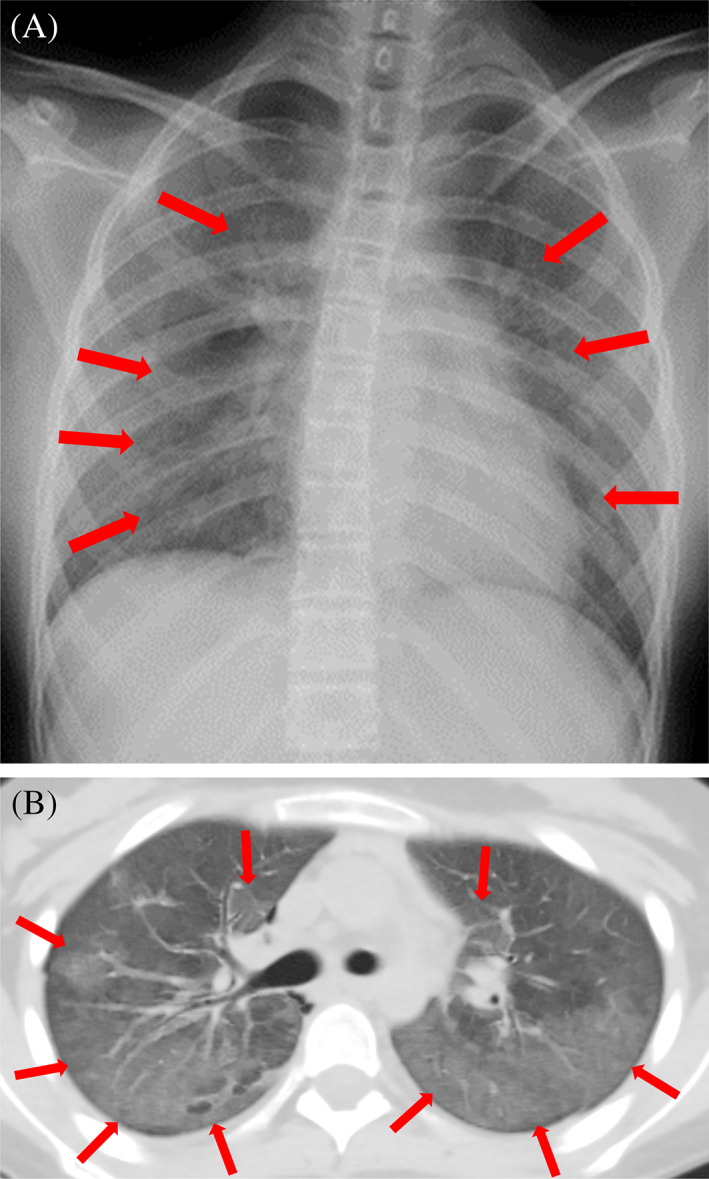
Imaging findings for a patient with *Pneumocystis jirovecii* pneumonia (PJP) after CD19‐targeted chimeric antigen receptor T‐cell therapy. (A) Chest radiography shows diffuse ground‐glass opacities indicated by red arrow marks in both lungs. (B) Chest computed tomography also shows diffuse ground‐glass opacities indicated by red arrow marks in both lungs, especially the dorsal regions. These findings are typical radiological features of PJP.

A 14‐year‐old girl was diagnosed with precursor B‐cell acute lymphoblastic leukemia (B‐ALL) at 7 years of age and treated per the protocol for the standard‐risk group in the Japanese Pediatric Leukemia/Lymphoma Study Group (JPLSG) ALL‐B12 clinical trial at another hospital in February 2015. Molecular remission was achieved at the end of the consolidation therapy. Two years after treatment completion, she developed combined relapse in the bone marrow and central nervous system. Molecular remission was achieved with multi‐agent and intrathecal chemotherapy, and umbilical cord blood transplantation (CBT) was performed 4 months after the diagnosis of relapse. The patient developed hemophagocytic lymphohistiocytosis (HLH) and exhibited delayed engraftment following CBT. Ten months after CBT, she developed autoimmune cytopenia with the production of anti‐neutrophil antibodies, anti‐erythrocyte antibodies, and platelet‐associated IgG (PA‐IgG), as well as pleural effusion and ascites. She was treated with prednisolone, cyclosporine, mycophenolate mofetil, and rituximab. Twenty‐one months after CBT, she presented with dyspnea, dysuria, diarrhea, and disorders of consciousness and was diagnosed with combined second relapse in the bone marrow and central nervous system. The ferritin level was 10 606 ng/mL at the second relapse. She underwent multidrug chemotherapy, intrathecal chemotherapy, and whole‐brain irradiation. Subsequently, she was referred to Keio University Hospital for CD19‐targeted CAR‐T cell therapy in September 2021, 1 month after diagnosis of the second relapse. CD19‐targeted CAR‐T cell therapy was performed, and 2.2 × 10^6^/kg/dose of CAR‐T cells were administered (Table S1). Three to seven days after CAR‐T cell therapy, the patient developed fever as grade 1 cytokine release syndrome. Pancytopenia requiring blood transfusion persisted for 2 months after CAR‐T cell therapy. The patient remained in remission after the therapy and continued to receive TMP/SMX for PJP prophylaxis. The CD4+ T‐cell counts remained above 200/μL from 3 months and above 500/μL from 6 months after CAR‐T cell therapy. Phytohemagglutinin (PHA)‐induced lymphocyte proliferation was normal. TMP/SMX therapy was discontinued 7 months after CAR‐T cell therapy. CD19+ B‐cell aplasia persisted, and IgG levels were maintained at 400–800 mg/dL with periodic immunoglobulin replacement therapy. Ten months after CAR‐T cell therapy, she presented to our hospital with fever, cough, and dyspnea for 5 days. On admission, her body temperature was 38.0°C, and her O_2_ saturation was 91% on room air. Laboratory tests showed the following: white blood cell count, 8.0 × 10^9^/L (normal range: 3.8–9.4 × 10^9^/L) [band neutrophils, 4%; segmented neutrophils, 66%; lymphocytes, 21%; atypical lymphocytes, 2%; monocytes, 6%; eosinophils, 0%; basophils, 1%; CD4+ T‐cell count, 771/μL; CD19+ B‐cell count, 0/μL]; hemoglobin, 124 g/L (normal range: 118–149 g/L); hematocrit, 0.387 L/L (normal range: 0.350–0.436 L/L); mean corpuscular volume, 100 fL (normal range: 79.5–96.5 fL); platelet count, 92 × 10^9^/L (normal range: 170–410 × 10^9^/L); albumin, 3.3 g/dL (normal range: 3.8–4.8 g/dL); C‐reactive protein, 1.24 mg/dL (normal range: 0–0.14 mg/dL); aspartate aminotransferase, 60 U/L (normal range: 13–28 U/L); alanine aminotransferase, 29 U/L (normal range: 9–29 U/L); lactate dehydrogenase, 535 U/L (normal range: 130–250 U/L); β‐D glucan, 511 pg/mL (normal range: 0–11 pg/mL); KL‐6, 643 U/mL (normal range: 0–500 U/mL); soluble IL‐2R, 2494 U/mL (normal range: 121–613 U/mL); ferritin, 1163 ng/mL (normal range: 8–129 ng/mL); and IgG, 244 mg/dL (normal range: 861–1747 mg/dL). Chest radiography and computed tomography (CT) revealed diffuse ground‐glass opacities in both lungs (Figure 1A,B). Polymerase chain reaction (PCR) testing of the sputum showed positivity for *Pj*. PJP was diagnosed on the basis of the PCR test results, high β‐D glucan and KL‐6 levels, and characteristic CT findings. She was treated with immunoglobulin (250 mg/kg/day) for hypogammaglobulinemia and TMP/SMX (15 mg/kg/day of trimethoprim) and prednisolone (1.5 mg/kg/day) for PJP; this resulted in rapid amelioration of her symptoms. Immunoglobulin was administered only once. TMP/SMX was discontinued after 21 days, and prednisolone was tapered by 0.5 mg/kg/day every 5 days for 15 days. After treatment, the patient continued to receive TMP/SMX (4 mg/kg/day of trimethoprim) twice a week for PJP prophylaxis, along with periodic immunoglobulin replacement therapy to maintain her IgG levels above 600 mg/dL. Ten months after the treatment, there was no recurrence of PJP or any other complications.

## 3 DISCUSSION

PJP after CAR‐T cell therapy is rare, with only nine adult cases reported thus far. These included five cases with a CD4+ T‐cell count of <200/μL at onset and four cases with unknown CD4+ T‐cell data. The majority of cases received inadequate TMP/SMX for prophylaxis or exhibited failure of CD4+ T‐cell recovery or impaired function (Table 1).^1^, ^10^, ^12^, ^13^, ^14^ In the present case, TMP/SMX was discontinued 7 months after CAR‐T cell therapy for post‐CBT relapse of B‐ALL under the conditions of a CD4+ T‐cell count of >500/μL and normal PHA‐induced lymphocyte proliferation. Three months after discontinuation, the patient developed PJP despite a sufficient CD4+ T‐cell count of 771/μL. It is possible that the total number of CD4+ T cells included those derived from CAR‐T cells. Although fluorescence activated cell sorter analysis was not performed in this case, it is expected that the CD4+ T cells derived from CAR‐T cells were only a part of the total count at 10 months after CAR‐T cell therapy.^15^ The role of CD4+ T cells derived from CD19‐targeted CAR‐T cells in immunity against PJP is not clear, but it is not expected to be significant because they are only a part of the total count. To our knowledge, this is the first case of PJP in a patient with a CD4+ T‐cell count of >200/μL after CD19‐targeted CAR‐T cell therapy. This case is important because the patient developed PJP despite administration of TMP/SMX for prophylaxis for the recommended period and the absence of CD4+ T‐cell recovery failure or dysfunction, unlike previous cases.^1^, ^10^, ^11^, ^12^, ^13^, ^14^ There are two possible reasons for the development of PJP despite a CD4+ T‐cell count of >200/μL in this patient.

**TABLE 1:** Literature review of PJP after CAR‐T cell therapy.

Case	Age	Dis	CD4+ T at OP (/μL)	PP (m)	EP to OP (m)	EC to OP (m)	RF	Outcome	References
1	Unk (21 to 76)	LBCL	<200	10 to 15	<3	12 to 15		Survive	Baird et al.^1^
2		LBCL	<200	3	1	4		Survive	
3		LBCL	<200	3	1	4		Death	
4	64	LBCL	Unk	Unk	Unk	4		Survive	Little et al.^10^
5	64	LBCL	Unk	9	4	13	20 mg HC daily, HGG	Survive	
6	63	LBCL	<200	13	2	15	HGG	Survive	
7	Unk	LBCL	44	5	4	9		Survive	Wudhikarn et al.^12^
8	Unk	ALL	Unk	0		1 to 3		Survive	Hill et al.^13^
9	Unk	LBCL	Unk	0		<1		Survive	Strati et al.^14^

Abbreviations: ALL, acute lymphoblastic leukemia; CD4+ T, CD4+ T cell counts; Dis, disease; EC, end of CAR‐T cell therapy; EP, end of prophylaxis; HC, hydrocortisone; HGG, hypogammaglobulinemia; LBCL, large B‐cell lymphoma; m, months; OP, onset of PJP; PP, prophylactic period; RF, risk factor; Unk, unknown.

First is the inadequate activation of CD4+ T cells owing to B‐cell depletion after CAR‐T cell therapy. In immunity against *Pj*, CD4+ T cells are generally considered important; recently, B cells have also been given importance in this regard.^3^, ^4^, ^5^, ^6^, ^7^, ^8^, ^9^ Antigen presentation by B cells to CD4+ T cells was found to be particularly important in PJP mouse models.^4^ The study showed that CD4+ T cells were not activated when B cells were depleted, even when sufficient dendritic cells were present as antigen‐presenting cells. It is also shown that IgG and IgM produced by B cells are involved in immunity against *Pj*; however, these factors alone are not sufficient.^4^ Our patient had a low IgG level of 244 mg/dL on admission, which may have contributed to the onset of PJP. However, the patient was seen several days after the onset of symptoms, and it is possible that her IgG level was consumptively low as a result of *Pj* infection. In the immune response against *Pj*, B cells present antigens to CD4+ T cells, which are mutually activated. Activated CD4+ T cells act against antigens by producing inflammatory cytokines and activating alveolar macrophages. In contrast, activated B cells produce antibodies specific for the *Pj* antigen and promote opsonization by macrophages.^16^ Since CD19‐targeted CAR‐T cell therapy results in B‐cell aplasia, the absence of antigen presentation from B cells results in the inactivation of CD4+ T cells; furthermore, no antibodies are produced by B cells. In this case, the CD4+ T cells were active because of normal PHA‐induced lymphocyte proliferation, but their activity specific to *Pj* antigen was probably reduced because of the absence of *Pj* antigen presentation from B cells.

The second reason is an abnormal macrophage response after CBT. Compared with allogeneic bone marrow transplantation, CBT requires approximately 1 week longer for engraftment, and patients are prone to engraftment failure and delayed engraftment as well as engraftment syndrome, in which fever, pleural effusion, ascites, and diarrhea occur due to cytokine storm before and after engraftment.^17^, ^18^, ^19^ HLH is also a common complication after CBT.^20^ Our patient developed HLH with bone marrow findings of hemophagocytic macrophages and delayed engraftment. Anti‐neutrophil antibodies, anti‐erythrocyte antibodies, and PA‐IgG produced during pancytopenia after engraftment are associated with autoimmune‐associated hemophagocytic syndrome (AAHS).^21^ The patient also had pleural effusion and ascites. These findings suggested that cytokine storm and abnormal activation of macrophages were present. Dyspnea, dysuria, diarrhea, impaired consciousness, and an abnormally high ferritin level of 10 606 ng/mL at the second relapse, as well as the prolonged pancytopenia after CAR‐T cell therapy, were also expected to be related to the cytokine storm and macrophage hyperactivation.^17^, ^22^, ^23^ At the onset of PJP, the patient had a high ferritin level, a high soluble IL‐2R level, elevated liver enzymes, thrombocytopenia, and hypoproteinemia, which were also probable indicators of an abnormal macrophage immune response, as observed in HLH.^24^ Although the M1/M2 phenotype of the macrophages was not examined, frequent occurrences of cytokine storm associated with abnormal macrophage activation were considered on the basis of her clinical course, high ferritin level, and high soluble IL‐2R level, among other factors. Immunity against *Pj* requires macrophages and cytokines, which can cause lung injury at the same time.^16^ Previous reports have documented the association between PJP and HLH^25^ as well as the association between HLH and lung injury.^26^ Late‐onset HLH after transplants is triggered by viral and fungal infections.^27^ In this case, *Pj* infection was thought to have triggered abnormal macrophage activation and cytokine storm, resulting in lung injury and symptomatic PJP.

Thus, the patient developed PJP despite a CD4+ T‐cell count of >200/μL because of a predisposition to abnormal macrophage activation and cytokine storm after CBT as well as B‐cell aplasia after CAR‐T cell therapy combined with decreased CD4+ T‐cell function specific for *Pj*. In patients predisposed to abnormal macrophage activation and cytokine storm, a risk of PJP complications should be considered even if CD4+ T cells recover under B‐cell depletion after CD19‐targeted CAR‐T cell therapy. In such cases, it may be better to continue TMP/SMX for prophylaxis until B‐cell recovery after CAR‐T cell therapy. It is important to determine the duration of TMP/SMX for prophylaxis after CAR‐T cell therapy according to each case as well as the CD4+ T‐cell count. However, this conclusion is based on the findings for a single case, and further studies are needed for a better understanding of the involved immune mechanisms and optimization of prophylactic strategies for patients undergoing CAR‐T cell therapy.

## AUTHOR CONTRIBUTIONS

**Kento Kawata:** Conceptualization (lead); data curation (lead); writing – original draft (lead); writing – review and editing (lead). **Haruko Shima:** Conceptualization (supporting); supervision (equal); writing – original draft (supporting); writing – review and editing (supporting). **Masayoshi Shinjoh:** Conceptualization (supporting); supervision (supporting); writing – original draft (supporting); writing – review and editing (supporting). **Fumito Yamazaki:** Data curation (supporting); writing – review and editing (supporting). **Takumi Kurosawa:** Data curation (supporting); writing – review and editing (supporting). **Mizuki Yaginuma:** Data curation (supporting); writing – review and editing (supporting). **Hiroshi Takada:** Data curation (supporting); writing – review and editing (supporting). **Hiroyuki Shimada:** Conceptualization (supporting); supervision (equal); writing – original draft (supporting); writing – review and editing (supporting).

## CONFLICT OF INTEREST STATEMENT

The authors have stated explicitly that there are no conflicts of interest in connection with this article.

## ETHICS STATEMENT

Written informed consent was obtained from the patient and her parent for the publication of this article and any accompanying images. This article does not include any information that required IRB approval.

## Supporting information


**Table S1.** Preparation and dosage of CAR‐T cells.

## Data Availability

Data sharing is not applicable to this article as no new datasets were created or analyzed in this study.
