# Supplementary material for: Pneumocystis jirovecii pneumonia after CD4+ T‐cell recovery subsequent to CD19‐targeted chimeric antigen receptor T‐cell therapy: A case report and brief review of literature
Source: Cancer Rep (Hoboken). 2023 Aug 10;6(10):e1885. doi: 10.1002/cnr2.1885 (PMC10598253; doi:10.1002/cnr2.1885)
Supplement: Supplementary file 1 — Table S1. Preparation and dosage of CAR‐T cells. [file CNR2-6-e1885-s001.docx]

**Table S1.** Preparation and dosage of CAR-T cells

| Leukapheresis | CD3+ lymphocyte counts of 3.15×10^9^  Total nucleated cell counts of 6.58×10^9^ |
| --- | --- |
| Lymphodepleting chemotherapy | Fludarabine 30 mg/m^2^ day 1-4  Cyclophosphamide 500 mg/m^2^ day 1-2 |
| CAR-T cell infusion | CAR-T cell counts of 2.2×10^6^ /kg/dose |
